# Consistent levels of A-to-I RNA editing across individuals in coding sequences and non-conserved Alu repeats

**DOI:** 10.1186/1471-2164-11-608

**Published:** 2010-10-28

**Authors:** Shoshana Greenberger, Erez Y Levanon, Nurit Paz-Yaacov, Aviv Barzilai, Michal Safran, Sivan Osenberg, Ninette Amariglio, Gideon Rechavi, Eli Eisenberg

**Affiliations:** 1 Vascular Biology Program and Department of Surgery, Children's Hospital Boston and Harvard Medical School, Boston, MA 02115, USA; 2 Department of Dermatology, Sheba Medical Center, Tel-Hashomer, Israel; 3 The Mina and Everard Goodman Faculty of Life Sciences, Bar-Ilan University, Ramat-Gan 52900, Israel; 4 Cancer Research Center, Sheba Medical Center, Tel-Ha'Shomer, and Tel-Aviv University, Israel; 5 Raymond and Beverly Sackler School of Physics and Astronomy, Tel-Aviv University, Tel Aviv 69978, Israel

## Abstract

**Background:**

Adenosine to inosine (A-to-I) RNA-editing is an essential post-transcriptional mechanism that occurs in numerous sites in the human transcriptome, mainly within Alu repeats. It has been shown to have consistent levels of editing across individuals in a few targets in the human brain and altered in several human pathologies. However, the variability across human individuals of editing levels in other tissues has not been studied so far.

**Results:**

Here, we analyzed 32 skin samples, looking at A-to-I editing level in three genes within coding sequences and in the *Alu *repeats of six different genes. We observed highly consistent editing levels across different individuals as well as across tissues, not only in coding targets but, surprisingly, also in the non evolutionary conserved *Alu *repeats.

**Conclusions:**

Our findings suggest that A-to-I RNA-editing of *Alu *elements is a tightly regulated process and, as such, might have been recruited in the course of primate evolution for post-transcriptional regulatory mechanisms.

## Background

Site-selective adenosine to inosine (A-to-I) RNA-editing is an essential post-transcriptional mechanism for expanding the proteomic repertoire. It is carried out by members of the double-stranded RNA-specific ADAR family predominantly acting on precursor messenger RNAs [1]. As inosines in mRNA are recognized as guanosines (G) by the ribosome in the course of translation, RNA-editing may lead to a codon exchange resulting in a modified protein. ADAR-mediated RNA editing is essential for the development and normal life of both invertebrates and vertebrates [2-5]. Additionally, altered editing patterns have been found to be associated with various diseases including inflammation [6], SLE [7], epilepsy [8], depression [9], ALS [10,11] and malignant brain tumors such as gliomas and astrocytomas [12-14]. A-to-I editing affects numerous sites in the human transcriptome, most of which are located in *Alu *elements within untranslated regions [15-19]. *Alu *is a retrotransposon, about 280 bp long, belonging to the class of Short Interspersed Nuclear Elements (SINEs). More than one million copies, are present in the human genome, comprising 10% of the whole genome mass [20]. The precise role of RNA editing in *Alu *repeats is yet a mystery. However, it might affect gene expression through a number of mechanisms [21]: As inosine pairs with cytosine, editing influence the stability of RNA molecules by creating and disrupting secondary structures. At another level, since inosine is recognized as guanosine by the splicing machinery A-I editing can lead to modification of splice sites in introns, inducing premature termination, frame-shift, or new exon formation [22,23].

Consistent levels of A-to-I RNA-editing across human individuals were previously observed in a few recoding sites, mainly glutamate receptors [10,12,24-26]. However, the variability of the editing level among different human individuals has so far not been studied for neither most recoding editing targets nor the abundant *Alu *editing. Low variance of the editing level indicates a tight regulation and might attest to the functional importance of the specific editing event.

To test this, we studied the variability in editing levels across different human individuals and a variety of tissues, genes and sites.

## Results and Discussion

### Editing levels are consistent among skin samples of human individuals both in coding and non-coding sequences

In healthy brain tissue, editing levels for the recoding sites within the glutamate receptor are highly uniform across individuals [10,12,24]. As a first step, we tested whether the same is true for other editing sites occurring within coding sequences expressed in other tissues.

First, we investigated 32 skin samples. Although mice ADAR1 or ADAR2 knockout die in-utero or shortly after birth, RNA editing is implicated as relevant to the skin in humans by the observation that mutations in ADAR1 lead to Dyschromatosis symmetrica hereditaria [27], a pigmenting genodermatosis with an autosomal dominant inheritance reported predominantly in Japanese and Chinese individuals [28]. It is interesting to note that the effect of these mutations on ADAR1 function or editing pattern has not been found.

**Figure 1:**
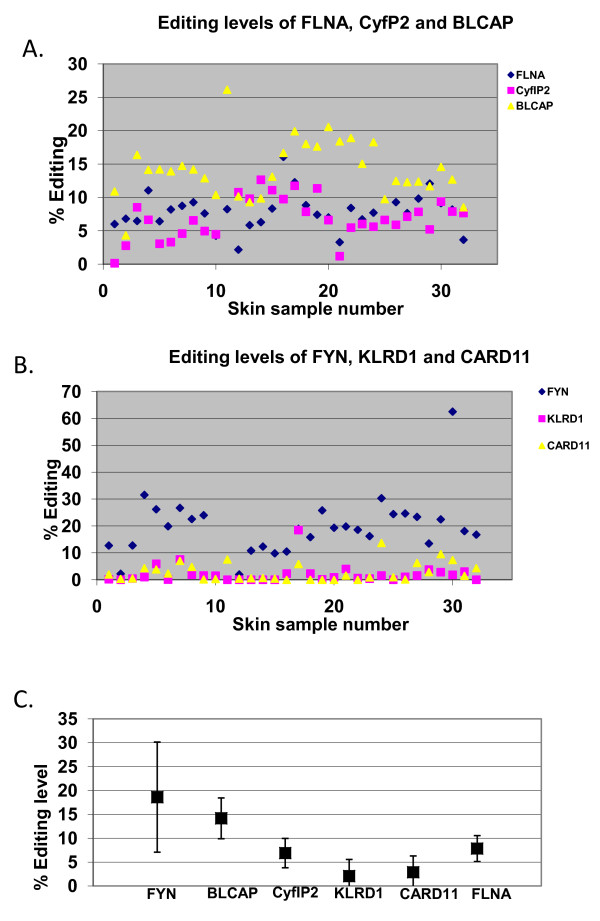
**Highly regulated A-to-I RNA-editing within coding sequences and *Alu *repeats in human skin samples**. 32 skin samples were tested for editing levels in one site within the coding regions of *FLNA*, *CYFIP2 *and *BLCAP *and within *Alu *repeat residing in the FYN, KLRD1 and CARD11 genes. **A**. Consistent editing levels in *FLNA*, *CYFIP2 *and *BLCAP *among different individuals. **B**. Consistent *Alu *A-to-I RNA-editing levels in *FYN*, *KLRD1 *and *CARD11*. **C**. Mean editing level of the six sites ± one standard deviation.

We looked at A-to-I editing levels in three recently discovered, mouse-conserved, targets within coding sequences: FLNA, CYFIP2 and BLCAP [29,30]. Filamin A (FLNA) displays an A→I editing site in its transcript (chromosome X:153,233,144, edited by both ADAR1 and ADAR2 [31] ), resulting in a Q→R substitution at amino acid 2341 in the human protein. The CYFIP2 (cytoplasmic FMR1 interacting protein 2) transcript encodes a protein of 1253 amino acids, and undergoes A→I editing (by ADAR2 [31]) at chromosome 5:156,669,386, resulting in K→E substitution at amino acid 320. BLCAP (bladder cancer associated protein), is highly conserved among species, having 91% and 100% identity at the DNA (coding region) and protein levels, and is recoded by editing. Here, we tested one editing site in the nucleotide encoding the second codon of BLCAP, located at chromosome 20:35,580,986, resulting in a Y→C substitution. Sequenome analysis for RNA editing [32] has shown an average editing level of 8, 9 and 14% for FLNA, CYFIP2 and BLCAP, respectively, in the skin samples. Remarkably, in all three genes, we observed a comparable level of editing for the 32 individuals, as mirrored by the low standard deviations - in all three sites the standard-deviation of the editing levels across individuals is about 1/3 of their averaged editing level (standard deviations were 2.7, 3 and 4.2 for FLNA, CYFIP2 and BLCAP, respectively, where editing levels are measured on a 0-100 scale as usual; see Figure 1A). These finding suggested that the protein diversity derived from editing is tightly regulated in these targets. Editing levels of FLNA and CYFIP2 in skin were lower than the values reported for normal brain tissue, while in BLCAP, the level was comparable to the one in normal brain tissue, oral cavity and lung [13]. This observation is consistent with previous reports suggesting that BLCAP is edited (almost) only by ADAR1, while the other two sites are edited by both ADARs or by ADAR2 alone [31].

Next, we tested the variability in editing levels of non-recoding sites. In particular, we focused on editing targets within the primate-specific *Alu *repeats which constitute the vast majority of editing sites in the human genome. In this category we looked at a single editing site in 3 targets selected from the RNA editing database [18]: FYN (last intron, chr6:112,094,677; (hg18)), CARD11 (the 14th intron, chr7:2,942,082), and KLRD1 (3' UTR, chr12:10,359,728). Using Sequenom analysis of the above 32 skin samples, we found low average editing levels in these sites, 19, 2, and 3% (with standard deviations of 11, 3 and 3) for FYN, KLRD1 and CARD11, respectively (Figure 1B, C). In order to quantify the degree of consistency among individuals, we compared the individual editing levels of the 6 different sites in the FLNA, CYFIP2, BLCAP, KLRD1, CARD11 and FYN genes. A statistically significant difference (p < 0.05 by Mann-Whitney test) between editing levels of the different sites was observed for 13 out of the 15 pairs of editing sites tested (see supplementary Table 1). We therefore conclude that the variation of results across individuals is significantly lower than the difference in editing efficiency between different sites (F-ratio 41.8 by ANOVA analysis; p-value 1.1E-28).

**Figure 2:**
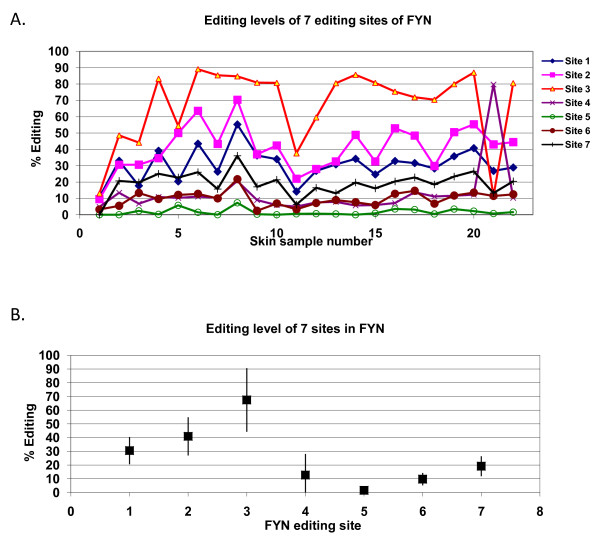
**Editing levels of specific sites in a highly-edited region of *FYN *is consistent among individual human skin tissues**. **A**. Editing levels for sites in a highly-edited region of *FYN *are presented for 22 skin samples. Site-specific editing levels are consistent among the samples. **B**. Editing level and variance of the seven tested sites of *FYN*. Mean editing level of the seven sites ± one standard deviation.

Most editing sites in the human transcriptome occur in clusters where a number of nearby sites undergo editing. Therefore, the question arises whether editing regulation occurs at the cluster level or at the site level. That is, whether regulation is able to distinct editing sites residing in the same highly-edited region. To answer this question, we direct sequenced an *Alu *repeat within the last intron of the FYN gene for 32 different human skin samples and analyzed 7 different editing sites in this region (see Additional file 1). Twenty two of the samples resulted in high-quality sequence data. Distinct editing levels were found for the 7 sites (Figure 2). Mann-Whitney analysis showed the editing levels of different sites to be distinct: 20 out of 21 comparisons resulted in a significant (p < 0.05, supplementary Table 2) difference. ANOVA analysis resulted in F-ratio 68.25 (p = 4.9E-40), demonstrating that the difference in editing efficiency among various sites in the same *Alu *repeat is an order of magnitude larger than variability in editing efficiency of specific sites across individuals.

### Comparison of editing levels in different tissues

It is established that editing levels are particularly high in some tissues, including the brain [33]. This can be attributed to the elevated level of ADAR2 in the brain. Here we aim at comparing the editing levels in a number of non-brain tissues. For this purpose we analyzed editing data [18] for clusters of editing sites in the 3'UTR *Alu *sequences of 3 different genes: MDM4, NRIP3 and THOC5 (see Additional file 1) in the following tissues: lung, kidney, prostate, uterus, liver and glioma tumor (not including normal brain tissue). We found low variability among different tissues and a clear distinction between different editing sites of the 3 genes. That is, given the six values of editing levels in the six non-brain tissues, for two different sites within the same Alu repeat, the tissue-to-tissue variance is low enough to allow one to tell (using a standard statistical test, e.g. t-test or Mann-Whitney) that the two sets of six measurements describe two sites differing in their editing efficiency. The tissue-to-tissue similarity is high enough to allow pair-wise distinction between different sites in a cluster in 51/91, 148/300 and 50/105 of the pairs, in MDM4, NRIP3 and THOC5, respectively (p < 0.05; Mann-Whitney test. supplementary tables 3-5). ANOVA analysis yielded F-ratios 12.71, 18.20 and 5.29 (p-values 7.5E-16, 1.4E-26 and 4.7E-07) for MDM4, NRIP3 and THOC5, respectively, again showing that the tissue-to-tissue variability is an order of magnitude lower than the site-to-site variability within the same cluster. The standard-deviation to mean ratio is 0.34, 0.49 and 0.62 for MDM4, NRIP3 and THOC5 (averaged over all sites in the same gene).

**Figure 3:**
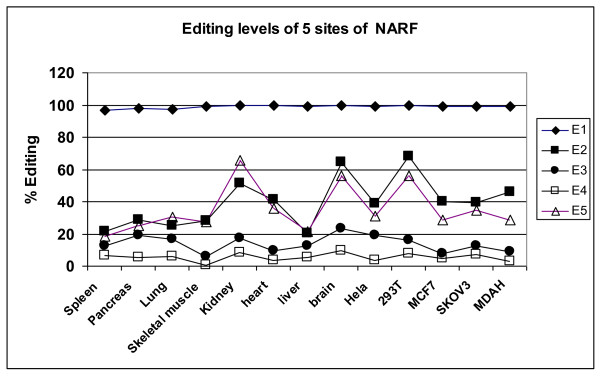
**Editing levels in the *NARF *gene are regulated among different tissues and cell-lines**. Data from Lev-Maor et al [34] was analyzed. Editing frequencies of five A-to-I editing sites within the *Alu *repeat in the 8^th ^exon of *NARF *are shown for 13 different tissues and cell-lines.

Regulated editing events might be recruited for functional processes. As an example, we studied the *Alu *editing in the *NARF *gene. In this gene, insertion of *Alu *retrotransposon pair into an intron led to editing of the *Alu *repeats, which in turn created a novel primate-specific alternatively spliced exon [34]. We looked at the tissue-to-tissue consistency of the editing levels in 5 different sites within this *Alu *using the data published by Lev-Maor et al [34]. In concordance with our previous data, the editing levels of NARF were highly consistent among different human tissues including transformed and cancerous human cell lines (Hela, 293T, MCF7, SKOV3 and MDA, Figure 3): statistically significant difference (p < 0.05) was observed in 9/10 comparisons (supplementary table 6). ANOVA analysis yields an F-ratio 182.6 (p = 7.39E-33), demonstrating once more the individual to individual scatter to be much lower than editing-efficiency variability.

## Discussion

Our findings demonstrate that editing levels display low variability among different human individuals not only in coding but also in the non-coding, non-conserved, *Alu *sequences.

RNA Editing of several evolutionary-conserved recoding sites is known to be of critical importance to proper cell development and function. Altered editing patterns in these sites are associated with severe phenotypes [4,8,35]. It is therefore not surprising to find that editing levels in these sites are uniform among individuals, suggesting tight regulation as one would expect. The three newly discovered recoding sites considered in this work are also extremely well evolutionary conserved, and it is thus reasonable to believe that they also have a critical role, yet to be explored. Consistently, we found that they also all have low variability of editing levels.

Virtually all A-to-I editing events in primates occur in the primate-specific *Alu *repeats, and are therefore not conserved among many species. The biological importance of these sites, if any, is yet to be shown. We therefore tested the consistency of editing levels in these sites in order to obtain a hint as to their potential role. Editing events of sites with highly variable levels among individuals are unlikely to serve as an important link in a chain of events being part of a biological pathway. The other side of the coin is that sites whose editing exhibit a consistent pattern in terms of editing levels might have been recruited in the course of evolution to serve a functional role. Surprisingly, we found that editing levels in sites with *Alu *repeats do present us with such consistent patterns. The variability among sites, including neighboring ones, is very high: where some sites show an extremely low level of a few percent, neighboring sites could be edited up to 60-80%. Yet, these seemingly fluctuating patterns are actually consistent among individuals - strong sites are strong in all samples and weak site are weak in all samples.

As all sites are edited by the same ADAR enzymes, what could be the mechanism providing the wide range of efficiencies on one hand, together with significant consistency on the other? We propose that A to I editing is subjected to two levels of control - ADAR expression and structural patterns of the dsRNA. The essential ADARs enzymes are expected to be tightly regulated to have consistent levels among different individuals. Indeed, it was found that editing enzymes are tightly regulated during development [25,36]. Here too, we found ADAR1 levels to be consistent, in most cases, between different individuals, supporting this assumption (see Additional file 1). Therefore, although the relation between editing levels and ADARs expression is probably not a simple linear one [12,25,31], it is likely that ADAR expression controls editing level. However, given a certain level of the ADAR enzymes, it seems likely that sequence and structural differences between sites determine their editing efficiency relative to other sites. The sequence and the resulting dsRNA structure formed by *Alu*, vary significantly from site to site, but are shared by all samples. Sequence analysis [37,38]of editing sites revealed a number of weak motifs. However, these alone cannot account for the observed tightly-regulated editing profiles. It is therefore plausible that structural motifs may take a role and should be analyzed as well.

The massive expansion of the *Alu *repeats in the primate genome has increased by order of magnitudes the amount of A-to-I editing in their transcriptome. The above analysis suggests that this phenomenon provided the primates with thousands of well-controlled and consistent transcriptomic "switches" that can be utilized for biological functions. It is not clear how many of these were actually adopted. One distinct example is the adoption of editing site in the NARF gene to create a new editing-assisted splicing event, resulting in a whole-new primate-specific alternatively-spliced exon [23]. The surprising consistency shown in editing of arbitrarily chosen sites within *Alu's *tantalizes us to wonder whether this mechanism of increasing diversification by creating new editing-assisted splicing events, could be more widespread. Of particular interest are miRNA target sites within *Alu *repeats [39,40], where one can clearly see how regulated control over single nucleotides might result in an efficient mechanism affecting the translation rate of the harboring gene.

## Conclusion

In summary, we show that editing events within *Alu *sequences exhibit a consistent pattern of editing levels across individuals. This might be attributed to sequence and structural motifs controlling the editing efficiency. Therefore, *Alu *intervention in the genome provides the primates with thousands of well controlled binary transcriptomic switches [41], available for use as additional regulatory mechanisms. Evidence for sporadic use of these switches already exists, but it is yet unclear how widespread this phenomenon is. As A-to-I editing is most abundant in the brain, the fascinating question than arises whether the above-mentioned mechanism might have played a role in primates' brain evolution.

## Methods

### Human skin tissues

The study was approved by the Institutional Helsinki Committee at Sheba Medical Center, Tel Hashomer, Israel and informed consent was properly obtained by all participants. Human skin tissues were frozen by liquid nitrogen after their removal at surgery and kept at -70°C until further use. Thirty two skin samples were tested; 20 inflammatory skin lesions with the following clinical and pathological diagnoses: Atopic/nummular Dermatitis n = 7, Drug eruption n = 2, Psoriasis n = 1, Allergic contact dermatitis n = 1 and Cutaneus T-cell Lymphoma (Mycosis Fungoides) n = 9. 12 Normal skin samples were collected at the Chaim Sheba Medical Center.

### RNA purification, reverse transcription (RT) and A-to-I RNA editing reading

Total RNA was isolated using TRIzol reagent (Invitrogen) according to the manufacturer's instructions. Random-primed cDNA synthesis was done on 2 μg of total RNA using M-MLV reverse transcriptase (Invitrogen) according to the manufacturer's instructions. For analyzing editing levels we used Sequenom (San Diego, CA) MassARRAY Compact Analyzer and MassARRAY Assay Design 2 software, as described before [32]. The primer sequences and reaction conditions are available as supplementary data (Additional file 1).

### Direct sequencing and A-to-I editing reading of *Alu*

In order to compare editing levels of different sites in a highly edited region within an *Alu *repeat, we direct sequenced the 3' UTR of FYN transcripts. PCR reaction for cDNA products was carried out using 100 ng (1 λ) of cDNA obtained as described above, at an annealing temperature of 60°C. The PCR product was separated by 1.5% agarose gel electrophoresis, and extracted using QiaQuick gel extraction kit (QIAGEN) and according to the manufacturer's instructions. cDNA sequencing was carried out in genetic analyzer 3100 (Applied Biosystems/Hitachi; Foster City, CA), and according to the manufacturer's sequencing protocol. High-quality sequences were analyzed. Sequencing results were read using Sequencher 4.2 software (1991-2004 Gene Codes Corp., Ann Arbor, MI); editing quantification was carried out using DS gene 1.5 software (Accelerys Inc. Discovery Studio 2003; San Diego, CA).

### Reference Human Genome version used in the research

Mar. 2006 (hg18) assembly

## Authors' contributions

SG participated in the study design, carried out the molecular genetic studies, performed the statistical analysis and participated in drafting the manuscript. EYL conceived the study, participated in its design and the bioinformatic analysis, and participated in drafting the manuscript. NPY helped with the sequencing and with the editing analysis, AB participated in collecting the skin samples and made the histological diagnoses, MS helped with the Sequenom experiments, SO carried out the Q-PCR component, NA and GR participated in the design and coordination of the experimental work, EE participated in the design of the study, guided the statistical analysis and participated in drafting the manuscript.

All authors read and approved the final manuscript.

## Supplementary Material

Additional file 1 **Supplementary information**. This file includes supplementary tables and figures, primers that have been used and detailed information about ADAR1 expression results. Click here for file
