# Supplementary material for: Consistent levels of A-to-I RNA editing across individuals in coding sequences and non-conserved Alu repeats
Source: BMC Genomics. 2010 Oct 28;11:608. doi: 10.1186/1471-2164-11-608 (PMC3091749; doi:10.1186/1471-2164-11-608)
Supplement: Additional file 1 — Supplementary information. This file includes supplementary tables and figures, primers that have been used and detailed information about ADAR1 expression results. [file 1471-2164-11-608-S1.PDF]

Supplementary information

Tight-regulation of A-to-I editing in human coding sequences and non-conserved *Alu* repeats.

Shoshana Greenberger[1,2*], Erez Y. Levanon[3*], Nurit Paz[4], Aviv Barzilai[2], Michal Safran[4], Sivan Aussenberg[4], Ninette Amariglio[4], Gideon Rechavi[4] and Eli Eisenberg[5]

1. PCR of FLNA, CYFIP2, BLCAP, KLRD1, CARD11 and FYN 1
2. Analysis of of multiple Editing targets in FYN *Alu* 3
3. ADAR Real-time quantitative PCR analysis 3
4. ADAR1 expression is consistent in the skin 4
5. Supplementary figure 1. 5
6. Supplementary table 1. 6
7. Supplementary Table 2. 7
8. Supplementary table 3. 8
9. Supplementary table 4 9
10. Supplementary table 5. 11
11. Supplementary table 6. 12
12. Supplementary figure 2. 12

1. PCR of FLNA, CYFIP2, BLCAP, KLRD1, CARD11 and FYN

The amplified regions were selected to be specific and unique. PCR amplification used Ready Mix PCR Master Mix (ABgene). The primer sequences are as follows:

BLCAP - 5' ACGTTGGATG-ATTAGGTCGGTTCCTGCAGC3' 3'ACGTTGGATG-AGGAGGACGGGCAGCA5' EXT- GCAGCCACTGGAGGCAA.

CYfP2 - 5' ACGTTGGATG-TTTCGGCGACATGCAGATAG3' 3'ACGTTGGATG-ACGTCCACTTGGACTTGTTC5' EXT- CTCTTCATAGTGAGCACTGGTCT **CARD11** -

5'ACGTTGGATG-AGATCACGCCACTGCACTC3' 3'ACGTTGGATG-CCAGGTCAAGCTTGCCTTTT5' EXT- CAGCCTGGTGACAGAGC

FLNA 5' ACGTTGGATG-TTCGTGGTGCCTGTGGCTT3' 3'ACGTTGGATG-TGGGCGGTTTCTCTCGGTG5' EXT- TCACTGTTTCTAGCCTTC

FYN 5' ACGTTGGATGTAGTAAGGTCTCGCTCTGTC3' 3' ACGTTGGATGGAAAGATTGCTTGAGCCCAG5' EXT- AGTGCAGTGGCACCATTTC

KLRD1 5' CGTTGGATGAAGGATTTGGACTGGGTGTG 3' 3'ACGTTGGATGCAAACTCCTGACCTCAAGTG5' EXT TGGTGGTTTATGCCTGT

All PCR reactions were carried out at an annealing temperature of 60°C.

2. Analysis of of multiple Editing targets in FYN *Alu*

In order to determine the editing levels in different editing sites within an hyper-edited region, the following FYN product was amplified. The 7 editing sited analyzed are marked. (chr6: 112094574-112094709; hg18)

TCACCCAGGCTGGAGTGCAGTGGCACCATTTCaGCTCaCTaCAGCCTCAACCTCCTGGGCTCAAGCAA

TCTTTCCACCTCAGCCTCCTGAaCaGCTGGGACCACAGGTaCATGCCACCATGCCCAGGTAATTTTTG

3. ADAR1 quantitative PCR analysis

Real-time Quantitative PCR was performed in order to determine the levels of mRNA expression of *ADAR1*; Primers were designed according to Primer-Express software guidelines (Applied Biosystems). Forward and reverse primers spanned introns to eliminate possible DNA contamination. Primers used were: 5’ACAGCCAAAGACACTCCCTCTC3’ 3’GGCTCAGCATGGCTATCTGG5’. Reactions were run on ABI 7900HT or ABI Prism 7000 sequence detection systems utilizing SDS 2.1 Software (Applied Biosystems). All reactions were run in duplicate or triplicate. Transcripts were detected using SYBR and were normalized to GAPDH..

4. ADAR1 expression is consistent in the skin

As most A-to-I editing is the result of ADAR1 activity, we tested the variability of ADAR1 expression in the skin samples. Two major species of ADAR1 have been reported: an IFN-inducible 150 kDa protein present in the cytoplasm and nucleus, and a second 110 kDa protein that is constitutively expressed and predominantly nuclear(Patterson & Samuel, 1995). The two species arise through the use of different promoters that initiate transcription at alternative sites in the first exon, so that the IFN-inducible form comprises a novel 300 amino acids in the amino terminus. ADAR1 is widely expressed in many tissues, including skin and has been shown to be involved in the editing of specific glutamate-gated ion channels (Patterson & Samuel, 1995)). Recently, we reported overall reduction in ADAR in brain tumors compared to normal brain tissues (Paz et al., 2007). Using quantitative real-time PCR we Analysed 17 skin samples (9 inflammatory lesions and 8 Cutaneus T-cell Lymphoma) for ADAR1 expression . We have found a consistent expression of ADAR1 in almost all the tested samples (see supplementary figure 1), suggesting a tight control of the A-to-I editing process.

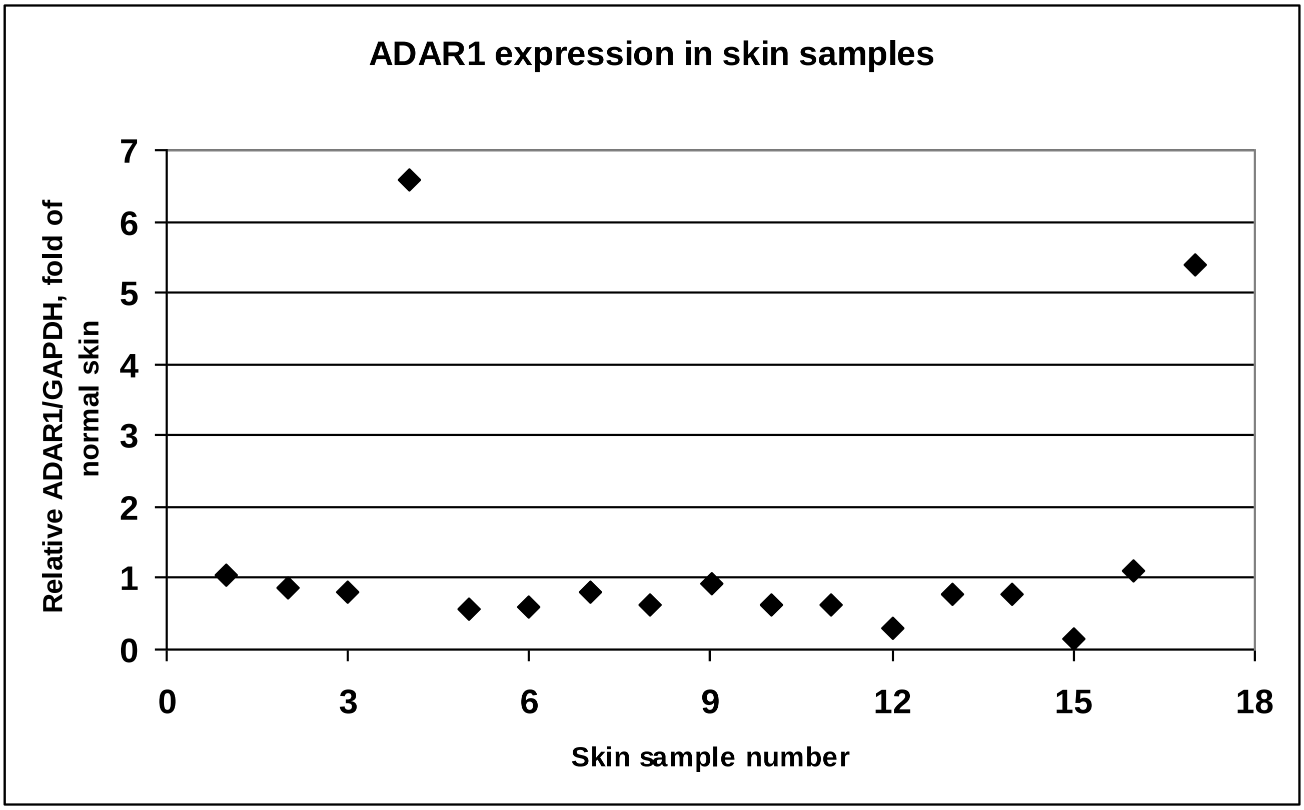

5. Supplementary figure 1.

Quantitative RT-PCR of ADAR1 mRNA expression in 17 skin samples.

	FYN	BLCAP	CyfIP2	KLRD1	CARD11
**FYN**					
**BLCAP**	2.13E-02				
**CyfIP2**	1.08E-07	9.54E-11			
**KLRD1**	6.33E-11	3.12E-14	6.45E-09		
**CARD11**	1.85E-10	1.28E-14	4.50E-06	2.06E-01	
**FLNA**	1.58E-07	1.25E-10	2.25E-01	8.28E-12	3.10E-08

6. Supplementary table 1.

Mann Whitney Statistical analysis of editing levels between FLNA, CYFIP2 , BLCAP, FYN, KLRD1 and CARD11.

In order to determine the level of consistency in editing frequency among the different skin samples, we compared the variance between the individuals to the variance between the editing levels of the different genes. P values are presented.. 13 out of 15 comparisons are statistically significant.

P Values

Site #	1	2	3	4	5	6
1						
2	5.07E-02					
3	2.01E-04	1.43E-03				
4	8.58E-05	1.21E-04	1.88E-06			
5	2.32E-11	1.49E-09	4.73E-11	2.96E-11		
6	6.33E-06	1.18E-05	6.45E-07	8.63E-01	6.45E-07	
7	1.08E-03	3.45E-05	1.76E-05	3.82E-03	1.76E-05	1.33E-03

7. Supplementary Table 2.

Mann Whitney Statistical analysis of editing levels among 7 editing sites of FYN. 19 out of 21 comparisons are statistically significant.

P Values

Site #	1	2	3	4	5	6	7	8	9	10	11	12	13
1													
2	**0.0011**												
3	0.7209	**0.0104**											
4	0.0650	**0.0002**	0.6454										
5	0.7984	**0.0002**	0.4418	**0.0011**									
6	0.1605	**0.0003**	0.1605	**0.0003**	0.0650								
7	0.3823	**0.0002**	0.8785	0.1304	**0.0207**	**0.002**							
8	**0.0499**	**0.0011**	0.1049	**0.0002**	**0.0006**	**0.038**	**0.000**						
9	0.8785	**0.0002**	0.7984	**0.0379**	0.5737	**0.050**	0.721	**0.001**					
10	0.9591	**0.0002**	0.6454	**0.0047**	0.9591	0.105	0.105	**0.001**	0.505				
11	0.1605	**0.0002**	0.9591	0.5054	**0.0148**	**0.002**	0.505	**0.000**	0.234	**0.038**			
12	0.7984	**0.0047**	0.4418	0.1304	0.8785	0.505	0.382	0.161	0.574	0.721	0.279		
13	**0.0030**	0.5737	**0.0148**	**0.0002**	**0.0002**	**0.002**	**0.000**	0.065	**0.000**	**0.001**	**0.000**	**0.015**	
14	**0.0006**	**0.0002**	0.5054	**0.0281**	**0.0002**	**0.000**	**0.003**	**0.000**	**0.002**	**0.000**	**0.007**	**0.005**	**0.000**

8. Supplementary table 3.

Mann Whitney Statistical analysis of editing levels in 14 sites of MDM4 3'UTR, among different tissues (n=8). 51 out of 91 comparisons are statistically significant.

P Values

Site#	1	2	3	4	5	6	7	8	9	10	11	12
**1**												
**2**	**0.008**											
**3**	0.222	0.056										
**4**	**0.008**	0.151	**0.008**									
**5**	**0.008**	0.548	**0.032**	0.310								
**6**	**0.008**	0.310	0.151	**0.032**	0.151							
**7**	**0.032**	0.548	0.310	**0.032**	0.421	1.000						
**8**	**0.008**	0.151	**0.008**	1.000	0.310	**0.032**	**0.032**					
**9**	0.690	**0.008**	0.310	**0.008**	**0.008**	**0.008**	0.056	**0.008**				
**10**	0.095	**0.016**	0.841	**0.008**	**0.016**	**0.032**	0.310	**0.008**	0.056			
**11**	0.095	0.056	1.000	**0.016**	0.056	0.310	0.310	**0.016**	0.151	0.690		
**12**	**0.008**	**0.032**	**0.008**	0.310	0.095	**0.008**	**0.008**	0.310	**0.008**	**0.008**	**0.008**	
**13**	0.310	0.310	0.841	**0.016**	0.151	0.421	0.690	**0.032**	0.222	1.000	1.000	**0.008**
**14**	**0.008**	0.222	0.421	**0.032**	0.421	1.000	1.000	**0.032**	**0.008**	0.095	0.095	**0.008**
**15**	**0.008**	**0.016**	0.548	**0.008**	0.056	0.222	0.421	**0.008**	**0.008**	0.310	0.548	**0.008**
**16**	**0.008**	0.690	**0.032**	0.310	0.841	0.151	0.310	0.421	**0.008**	**0.016**	**0.032**	0.095
**17**	**0.008**	**0.008**	**0.008**	**0.016**	**0.008**	**0.008**	**0.008**	**0.032**	**0.008**	**0.008**	**0.008**	0.151
**18**	**0.008**	0.151	**0.008**	1.000	0.222	**0.032**	**0.032**	1.000	**0.008**	**0.008**	**0.016**	0.548
**19**	**0.008**	0.222	0.222	0.095	0.222	0.841	0.841	0.056	**0.008**	0.095	0.222	**0.032**
**20**	**0.008**	0.056	**0.008**	0.841	0.151	**0.008**	**0.008**	0.690	**0.008**	**0.008**	**0.008**	0.310
**21**	**0.008**	**0.008**	**0.008**	**0.008**	**0.008**	**0.008**	**0.008**	**0.008**	**0.008**	**0.008**	**0.008**	**0.008**
**22**	**0.032**	0.548	0.421	0.222	0.548	0.841	0.841	0.222	**0.032**	0.548	0.548	0.095
**23**	**0.008**	**0.032**	**0.008**	0.222	0.095	**0.016**	**0.032**	0.222	**0.008**	**0.008**	**0.016**	0.690
**24**	**0.008**	0.310	**0.016**	0.841	0.310	**0.032**	**0.032**	1.000	**0.008**	**0.008**	**0.016**	0.222
**25**	**0.008**	0.548	**0.032**	0.310	0.548	0.056	0.151	0.548	**0.008**	**0.016**	**0.016**	0.095

Site#	13	14	15	16	17	18	19	20	21	22	23	24
**13**												
**14**	0.421											
**15**	0.690	0.151										
**16**	0.151	0.222	**0.016**									
**17**	**0.008**	**0.008**	**0.008**	0.151								
**18**	**0.032**	**0.032**	**0.008**	0.310	**0.032**							
**19**	0.421	1.000	0.310	0.222	**0.008**	0.056						
**20**	**0.008**	**0.032**	**0.008**	0.151	**0.008**	0.841	0.056					
**21**	**0.008**	**0.008**	**0.008**	**0.008**	**0.008**	**0.008**	**0.008**	**0.008**				
**22**	0.421	0.548	1.000	0.310	**0.016**	0.222	0.841	0.151	**0.008**			
**23**	**0.016**	**0.016**	**0.008**	0.222	0.310	0.421	**0.016**	0.222	**0.008**	0.095		
**24**	0.056	0.056	**0.008**	0.690	**0.016**	0.690	0.056	0.421	**0.008**	0.421	0.222	
**25**	0.056	0.222	**0.016**	0.841	**0.008**	0.222	0.222	0.222	**0.008**	0.421	0.222	0.690

9. Supplementary table 4.

Mann Whitney Statistical analysis of editing levels in 25 sites of NRIP3 3'UTR, among different tissues (n=5). 148 out of 300 comparisons are statistically significant.

Site #	1	2	3	4	5	6	7	8	9	10	11	12	13	14
**1**														
**2**	0.421													
**3**	**0.008**	**0.008**												
**4**	1.000	1.000	**0.008**											
**5**	0.056	0.056	**0.008**	**0.016**										
**6**	1.000	1.000	**0.008**	0.548	**0.032**									
**7**	**0.008**	**0.008**	**0.008**	**0.008**	0.548	**0.008**								
**8**	**0.008**	**0.008**	0.056	**0.008**	0.056	**0.008**	0.151							
**9**	**0.008**	**0.008**	0.690	**0.008**	**0.016**	**0.008**	**0.032**	0.222						
**10**	0.222	0.421	**0.008**	0.421	0.095	**0.008**	**0.016**	**0.008**	**0.008**					
**11**	1.000	1.000	**0.008**	0.548	0.095	0.310	**0.016**	**0.008**	**0.008**	0.421				
**12**	0.421	0.690	**0.008**	0.310	0.421	0.690	0.151	**0.032**	**0.016**	0.841	0.421			
**13**	0.690	1.000	**0.016**	0.310	0.151	0.421	0.151	0.056	**0.032**	0.421	0.421	0.421		
**14**	**0.008**	**0.016**	0.222	**0.016**	0.151	1.000	0.310	1.000	0.690	**0.032**	**0.016**	0.056	0.056	
**15**	**0.036**	**0.036**	0.786	**0.036**	**0.036**	**0.008**	**0.036**	0.571	0.786	**0.036**	**0.036**	**0.036**	0.143	0.571

Table 4

P Values

10. Supplementary table 5.

Mann Whitney Statistical analysis of editing levels in 15 sites of THOC5 3'UTR, among different tissues (n=5). 50 out of 105 comparisons are statistically significant.

P Values

Site #	E1	E2	E3	E4	E5
E1					
E2	1.92E-07				
E3	1.92E-07	1.92E-07			
E4	1.92E-07	1.92E-07	1.92E-07		
E5	1.92E-07	0.51	1.92E-07	1.92E-07	

11. Supplementary table 6.

Mann Whitney Statistical analysis of editing levels in 5 sites of NARF, among different tissues and cell lines (n=13). 9 out of 10 comparisons are statistically significant

12. Supplementary figure 2.

A-to-I editing levels of non-coding regions is similar among different tissues.

Editing levels for highly-edited regions in 6 human tissues: lung, glioma, prostate, kidney, uterus and liver.

A. Editing levels of 14 sites of MDM4. Dots represent mean editing levels (n=8) ± standard deviation.

B. Editing levels of 25 sites of NRIP3. Dots represent mean editing levels (n=5) ± standard deviation.

C. Editing levels of 15 sites of THOC5. Dots represent mean editing levels (n=5) ± standard deviation

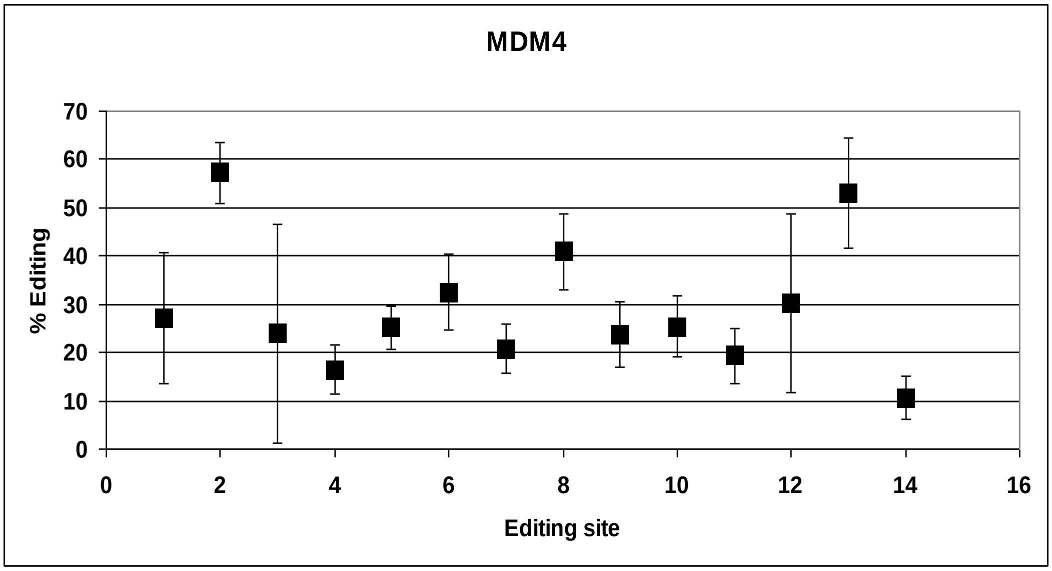
MDM4
% Editing
Editing site
70
60
50
40
30
20
10
0
0
2
4
6
8
10
12
14
16

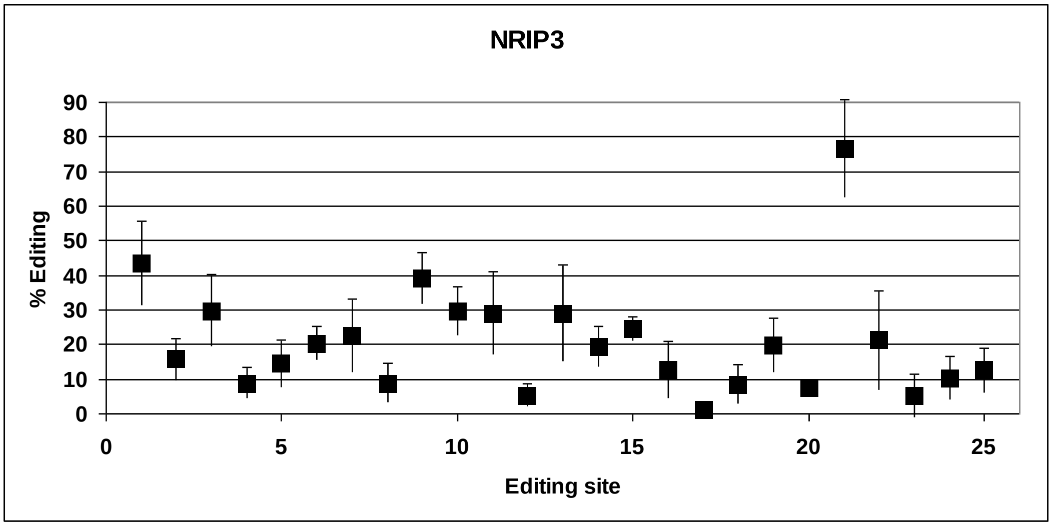
NRIP3
% Editing
Editing site
90
80
70
60
50
40
30
20
10
0
0
5
10
15
20
25

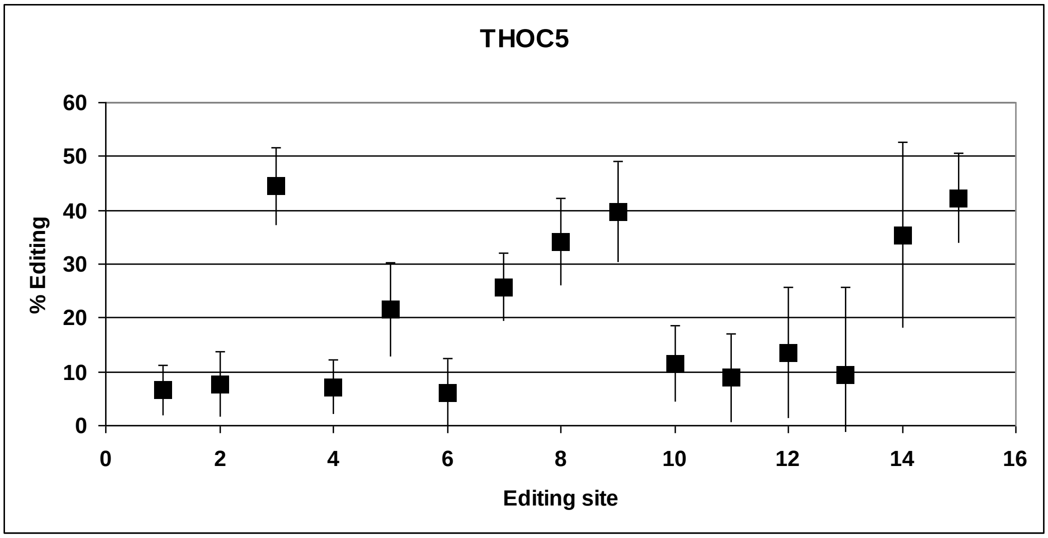
THOC5
% Editing
Editing site
60
50
40
30
20
10
0
0
2
4
6
8
10
12
14
16
